# Housing and Support Narratives of People Experiencing Mental Health Issues: Making My Place, My Home

**DOI:** 10.3389/fpsyt.2019.00939

**Published:** 2020-01-10

**Authors:** Ellie Fossey, Carol Harvey, Fiona McDermott

**Affiliations:** ^1^Department of Occupational Therapy, Monash University, Melbourne, VIC, Australia; ^2^Psychosocial Research Centre, Department of Psychiatry, University of Melbourne, Melbourne, VIC, Australia; ^3^NorthWestern Mental Health, Melbourne, VIC, Australia; ^4^Department of Social Work, Monash University, Melbourne, VIC, Australia

**Keywords:** housing, neighbourhood, mental illness, lived experience, qualitative research

## Abstract

**Background:** Choice, control, privacy, and security are widely reported housing preferences of mental health consumers, are associated with improved well-being and greater housing satisfaction, and are important for recovery. This paper describes housing and neighborhood experiences from a larger qualitative study that sought to learn with people experiencing mental health issues about their everyday lives in an Australian urban community.

**Methods:** A participatory approach to health research informed this study. A participatory reference group, including four people with consumer perspective knowledge and experience of mental health issues and four mental health practitioners with service provider and researcher perspectives, worked together to design and implement this study over a 4-year period. Thirty-nine participants were recruited, including 18 women and 21 men living in metropolitan Melbourne and receiving community mental health care for ongoing mental health issues related mainly to either psychotic or affective disorders. Participants each took part in one to three interviews or a focus group. The data were transcribed and analyzed using narrative and thematic analytic strategies, underpinned by reflective discussions with the participatory reference group.

**Findings:** Participants’ experiences of their housing and neighborhoods emphasized qualities that either contributed to or challenged their sense of being “at home.” Identifying with a place as home was transformative, especially when supported by friendly neighborhood interactions, safety, and accessibility of local amenities. Unsatisfactory housing situations and limited income worked against participants’ efforts to regain a sense of well-being and improve their situations. When being home was challenging, strategies used to counteract this included getting a pet and getting out as a means of resisting isolation at home. Differing views and ways of using the available support workers were described, suggesting tensions between seeking to be self-sufficient and valuing support.

**Conclusions:** Social housing locations and housing-related support should explicitly attend to safety and security concerns. Collaborative care planning and outreach support should attend to supports for navigating issues with neighbors, housing, harnessing natural supports, and opportunities for being in others’ company, as well as recognizing the importance of pets in people’s lives. Understanding the strategies that mental health consumers find helpful in creating a sense of being at home, and the role of “place” in recovery merit further consideration in research and practice.

## Introduction

Secure and appropriate housing is essential to the well-being of people living with mental health issues (1, 2) and contributes to the process of recovering (3), as well as being a basic human right (4). Historically, housing and support services for people living with persistent mental health issues began with the development of community-based residential alternatives to institutional care, typically characterized by communal living and on-site staffing, with aims of fostering housing stability and reducing hospitalizations (5). Beyond ordinary housing in an apartment or house with family or friends, different types of housing and support services have evolved to meet the needs of people experiencing mental illness internationally, and the terms used to describe them vary considerably between settings and countries (5–7). For instance, supported housing may describe supervised housing arrangements with staff support linked to the accommodation, such as group homes and communal residences (8). Yet, the term supported accommodation may also be used to differentiate that the support is provided by non-professional support staff, rather than clinically focused, irrespective of whether the housing is single or shared (6). In comparison, housing with outreach support describes an approach in which a person’s ongoing housing tenancy is de-coupled from the provision of treatment and individualized, flexible outreach support is emphasized (9). This approach is described variously as a Housing First, permanent supported or supportive housing approach (9, 10). In this paper, housing with outreach support describes the latter approach, and the term supported group accommodation is used for any group accommodation where there are staff on-site (11).

Housing research, predominantly from North America and the United Kingdom, has previously focused on housing characteristics, housing preferences, mental health, and psychosocial outcomes for people experiencing mental illness (12). Having choice and control of one’s living arrangements is a consistent theme across international studies of consumers’ housing preferences (7). Furthermore, choice in housing, residential stability, and neighborhood qualities, such as safety, appear to be associated with improved well-being and greater housing satisfaction (11, 13). The strongest evidence demonstrates the effectiveness of permanent housing with outreach support for formerly homeless people living with persistent mental illness, whereas evidence is less well developed regarding the effectiveness of supported housing of all kinds for other people experiencing mental illness (12, 14). Psychosocial outcomes across housing models ranging from independent tenancies with outreach support to supported group accommodation also remain difficult to compare due to the diversity in how these approaches have evolved and because conducting trials in this area is challenging (5, 12, 15). Yet, having a home and supports appears to reduce the likelihood of being hospitalized (12, 16). Improvements in social integration and recovery are also reported but less clear, but there may also be a greater risk of loneliness and isolation for residents living by themselves in housing with outreach support, albeit that results are mixed (2, 11).

Mental health consumer views and experiences of various types of housing and support have been explored through qualitative research. Recent reviews have synthesized the findings of over 60 qualitative studies exploring consumer experiences and perspectives of supported accommodation services (6); getting and having a home, and receiving housing support (10, 17); and how housing with outreach support facilitates social connections and participation (2). There is considerable overlap between these three reviews (reported in four papers) in terms of the studies included and the range of personal, social, and service-related factors identified that shape lived experiences of housing and support services. For instance, consistent with aforementioned studies of housing preferences, these reviews highlighted that consumers personally valued privacy, choice, and stable housing (2, 6, 17). Whether in permanent or transitional housing, having a home was central to consumers’ experiences of stability and thriving, connecting with others, and negotiating a positive sense of identity beyond that of being ill; their views also have much in common with more widely held meanings of home (6, 17). All three reviews also noted lived experiences of loneliness and isolation across individual and communal living situations, which required the balancing of needs for refuge, solitude, and social contact (2). Service factors like being required to move on from one housing service to another, becoming displaced, and losing social networks in the process exacerbated these experiences of loneliness and isolation (6). Yet, valued elements of support from services included individually tailored support, respectful and supportive relationships, assistance with practical matters and organizing activities, and neighborhood and community experiences that fostered inclusion, rather than reinforcing social isolation or exclusion (2, 10). This suggests consumer perspectives of their neighborhoods and communities, not only their housing, merit further exploration to better understand how to design support in different housing settings.

This paper reports findings from a participatory research project, using qualitative methods, undertaken with people experiencing mental health issues and living in a metropolitan region of Australia (18). The project aimed to learn what the everyday lives of people experiencing ongoing mental health issues are like, and to better understand community participation from their perspectives. This report focuses on one aspect of the larger project, that is, the housing and neighborhood experiences integral to participants’ everyday lives.

## Materials and Methods

### Design

This research was informed by a participatory approach to health research, in which the role of participation, the uses and construction of knowledge, an action orientation, and issues of power were considered in its design and conduct (19, 20). What makes research “participatory” has to do with who participates, the ways in which people are engaged in the research process, the spheres of their involvement, and whose purposes are served through participation (20). This study was designed to be *participatory* in two senses: firstly, through engaging people as partners in exploring their knowledge and understandings of their everyday lives and worlds; and secondly, through designing the research process to involve people acting *for* themselves and *with* others in a collaborative manner, rather than solely as the subjects of research (21).

Participatory approaches have been variously used in mental health research to address the needs of under-served populations, bring about systems change in mental health services, and better understand processes for supporting community re-integration and recovery (22–26). Drawing on principles outlined by Nelson et al. and Wadsworth and Epstein, a participatory reference group was convened as the key vehicle to amplify the voices of “critical” groups of people in representing their own interests and values in the research, and to determine the descriptions of themselves and their worlds that were used. This involved identifying and inviting people to take part in the reference group whose interests are “critical” to the research in question, and are the sources of “literally critical things to say about it” (26, p.56). Specifically, the membership was based on three main aims: to involve at least as many mental health consumers as people with other perspectives; to connect with consumer networks knowledgeable of the situations faced by consumers; and to foster dialogue across differing interests and knowledge bases (24, 27). Thus, members of the consumer advisory groups of local mental health services were invited to join the participatory reference group, and each of the mental health services was invited to nominate a representative. The resulting participatory reference group included four people with lived experience and consumer perspective knowledge of mental health issues and four members with mental health practitioner, carer, and researcher perspectives. This group worked together, meeting every 6–8 weeks over a 4-year period. The group defined the study focus, developed the recruitment strategies and qualitative interview guides, obtained feedback on the research processes, guided the qualitative analysis of interview data and development of themes, and presented the research at conferences over the course of the project.

### Setting

This research was undertaken in northern metropolitan Melbourne in south-eastern Australia, through the local mental health services that provide clinical care and community support for people with severe and persistent mental illnesses. Approximately 20% of Melbourne’s population lives in this geographic area, which extends from Melbourne’s inner city suburbs to its northern urban–rural fringe, with a highly culturally and linguistically diverse population and household incomes that tend to be lower than the metropolitan average (28).

### Sampling and Recruitment

Qualitative sampling aims to achieve sufficient sampling of information sources (i.e., people, places, events, types of data) to develop a full description of the phenomenon under study (29, 30). Adults of working age with lived experiences of mental health issues, resident in northern metropolitan Melbourne, and in receipt of community mental health services, were invited to participate in this project. Through purposive sampling, diversity in experiences in terms of age, gender, family structures, and educational and employment backgrounds were sought, so as to enhance the completeness of information gathered and to guard against privileging a particular perspective over others, issues central to rigor in qualitative sampling (31). To achieve this, the participatory reference group identified community mental health programs through which to approach people with relevant experiences, and negotiated locally responsive strategies with each program. Wherever possible, recruitment involved sharing information directly with mental health consumers, rather than relying on staff as intermediaries to distribute information. These strategies included the development of an information flyer for distribution at existing peer support groups, as well as attending meetings of established consumer advisory groups, social and recreational programs, and community rehabilitation and support programs that provide services for people experiencing ongoing mental health issues. As recruitment progressed, the evolving range of participants and experiences were discussed in participatory reference group meetings and additional sources chosen to extend our understanding of emerging issues. Written informed consent was completed with each participant, and all participants received remuneration (AUD$25) for each interview in recognition of their contribution and to limit out-of-pocket expenses related to participation.

### Participants

Thirty-nine participants took part in this research. As shown in **Table 1**, there were 18 women and 21 men, the majority of whom were between 30 and 49 years of age. While four participants (almost 10%) were employed full-time and 19 participants (49%) had some part-time or occasional paid work, government income support was the main source of income for most participants (70%). Participants reported experiencing mental health issues related to psychotic disorders (41%), bipolar disorder (28%), and depression and/or anxiety-related disorders (33%); almost half the participants (49%) had experienced and sought help for mental health issues for more than 10 years. In terms of housing, almost half of the participants lived with family, partners, or friends, and had resided in their present housing for 5 years or more, while among participants living in rental accommodation, seven (43%) identified this as public housing. The majority of participants received outreach support. Using the Simple Taxonomy for Supported Accommodation (STAX-SA) (32), this type of housing support is best classified as Type 4 (i.e., individual accommodation, no on-site staff, low/moderate support, and limited emphasis to move on—beyond that of market rental conditions).

**Table 1:** Socio-demographic profile of participants (N = 39).

		WOMEN n = 18	MEN n = 21	TOTAL N = 39
**Age**				
	18–29	1	6	7
	30–39	6	6	12
	40–49	7	6	13
	50–59	2	2	4
	60–65	2	1	3
Our relationships*(1)				
	Single	5	15	20
	Partner/married	9	4	13
	Separated/divorced/widowed	4	1	5
	Children	9	2 *(4)	11
Our homes*(4)				
	Family home	9	10	19
	Rented accommodation	9	7	16
Who we live with*(4)				
	Family with children	7	1	8
	Parents, partners, friends	5	6	11
	By self	6	10	16
	*Self with pets*	*5*	*2*	*7*
How long we have lived here*(4)				
	Less than 2 years	3	4	7
	2–5 years	5	4	9
	5–10 years	5	2	7
	Over 10 years	5	7	12
Experiencing and seeking help for mental health issues*(3)				
	2–5 years (typically “longer undiagnosed/longer without help”)	3	4	7
	5–10 years	4	6	10
	More than 10 years	11	8	19
Our education/training*(4)				
	University-level course	8	4	12
	Apprenticeship/vocational course	4	8	12
	High school only	6	5	11
**Our work**				
	Full-time paid work	2	2	4
	Part-time pad work (> 15 h)	5	10	15
	Casual/occasional paid work	3	1	4
	No paid work	8	8	16
	Unpaid volunteer	7	5	12

*() indicates no. of men for whom data is missing.

### Qualitative Data Collection

Multiple methods were used to collect information from different sources and perspectives, including in-depth interviews, follow-up reflections on participants’ stories, field notes, and recorded participatory reference group discussions. By illuminating different facets of participants’ experiences, this aimed to contribute to a more critical and complex understanding of their experiences as a whole (30).

In-depth interviewing was used to converse with participants about their everyday lives and participation in their communities, and to explore the contextual nature of these experiences (33). The participatory reference group developed an interview guide by beginning with a brainstorming activity on the topic of “our experiences of finding things to do in our communities,” followed by discussions that led to the identification of key content areas and phrasing for questions (see **Table 2**). Viewed as events in which meanings are negotiated, the in-depth interviews were constructed to include open-ended questions that established the topics being explored, and to support participants to take the lead in telling their stories, rather than the researcher directing the interview.

**Table 2:** Interview topics.

How do you spend your time at the moment…
Where do you spend time? Home/elsewhere?
What kinds of things do you do…
• For fun/enjoyment
• For quiet time—time out/to get away
• Working—paid, unpaid/voluntary
• Learning—study/classes for interest/education
• Around the house—chores/pets/helping others
• To be with other people for company, friendship, entertainment
What is important/matters to you in your life? Now?/Times when it’s been different? In what ways?
What’s been helpful/supportive in getting to do what matters to you?
• Places to go? Transport? Money? Information? People’s attitudes? skills?
• What’s been difficult/challenging/created obstacles for you related to doing these things?
What would you like to be doing in the future—dreams, hopes
• If you could wave a magic wand/if you could be doing whatever you choose, what would it be?
• What challenges/issues/fears would this involve overcoming?
• What might make it happen? What could help? In what ways?
Is there anything else that we have not covered that you think is important/would like to tell me about?

Interviews with the first author (an occupational therapist who previously worked in mental health services) took place at locations to suit participants as far as possible. Twenty-three participants preferred interviews at their homes, and on three occasions, participants’ partners also participated in the interviews. Sixteen participants chose to take part in interviews or a focus group at the research facility or their place of daytime occupation. The focus group was co-facilitated by the first author with a consumer researcher. The interviews and the focus group were digitally recorded or, when participants preferred, handwritten notes were made. Notes were elaborated immediately after interviews in as much detail as possible, and interview and focus group recordings transcribed verbatim. Each participant was sent their typed-up interview or focus group. Interview participants were invited to a follow-up interview with the first author, so as to create an opportunity for reflecting together with participants on what was said and understood, and to actively engage participants in interpreting their stories. As a result, the 39 participants took part in either 1 focus group or 1–3 interviews each: 54 interviews in total.

### Qualitative Data Analysis

Narrative and thematic analytic strategies were used. Interview transcripts and field notes were reviewed and coded using NVivo software, first by coding for meaning with “*in vivo*” codes that closely reflected participants’ language (34). Second, coding for narrative features, such as turning points, metaphors, and transformative elements, as well as attending to how power was revealed across their stories sought to uncover taken-for-granted assumptions or social structures affecting participants’ lives (35). As shown in **Table 3**, these steps were interwoven with going back and forth in an iterative manner between working with the data and discussions in participatory reference group meetings to inform the development of the themes.

**Table 3:** Steps undertaken to develop themes.

a) Transcribing the interviews;
b) “Mapping” each person’s interview story, through re-listening to the interview recordings, reviewing the transcripts and field notes to get to know the stories well;
c) Reflecting with individual participants on the “story maps” in follow-up interviews to share provisional understandings and create dialogue about their interpretation;
d) Developing a group process with participatory reference group for thematic analysis of the data;
e) Reviewing the story maps in the participatory reference group to identify preliminary themes;
f) Returning to the data to code and explore it, informed by the participatory reference group perspective;
g) Piecing together themes by working between writing, reviewing coded data, field notes, and recorded discussions with the participatory reference group;
h) Critical reflections on themes with the participatory reference group and feedback sought from local consumer groups.

## Findings

**Figure 1:**
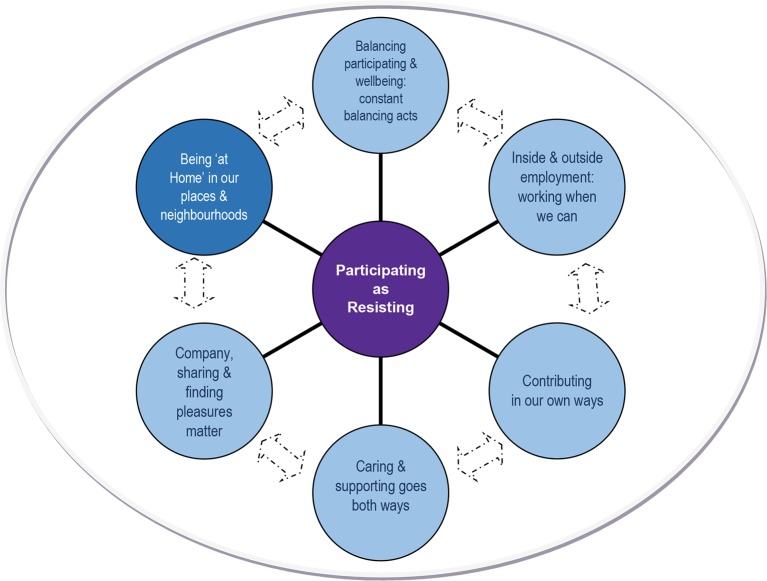
Diagrammatic representation of participating as “resisting” [Adapted from Fossey, 2009 (18)].

As a whole, this research revealed stories of ongoing struggles in everyday life that involved actively and intentionally striving to participate, to be oneself, and to be recognized as contributing by others in one’s community, which could not be taken for granted by participants. Hence, their stories evoked acts of resisting in a lived struggle to reclaim power within daily life, and their strategies for doing so. Six major themes were developed to account for these diverse ongoing struggles, ways of participating, and the social and material conditions revealed in participants’ stories, as illustrated in **Figure 1**. One of these themes—*being at home in our places and neighborhoods*—related specifically to participants’ experiences of housing and the immediate neighborhoods in which they live. Findings from this theme are described below, with participants’ voices integrated into the descriptive text using direct quotations and use of pseudonyms agreed upon with participants themselves.

Central to participants’ experiences of their housing and immediate neighborhoods were qualities that either contributed to or challenged their sense of being “at home” where they lived, impacts on their daily lives, and strategies that participants used to make a difference to home life. **Table 4** summarizes the sub-themes and categories presented below.

**Table 4:** Being “at home” in our places and neighborhoods: summary description.

Sub-theme	Category
My Place, My Home	A place of my own It’s good around here: finding my niche Everything’s accessible here
It’s stressful living here	There’s few options There’s poverty and there’s powerlessness
Being home is challenging	It’s better being out It’s not four walls
Balancing self-determination and need for support	

### My Place, My Home

*My Place, My Home* represents participants’ varied perspectives of home. Firstly, identifying with a place as home was appreciated as transforming participants’ lives, or restoring a sense of home lost while experiencing mental health issues. The freedom and privacy of living in a place of their own greatly improved their lives from these participants’ perspectives. Secondly, specific qualities of their immediate neighborhoods, notably friendly interactions, safety in the vicinity, and accessibility of local amenities, contributed to participants feeling at home where they lived and their sense of well-being. Each is illustrated.

#### A Place of My Own

Living in or moving to a place of one’s own, whether that meant living by oneself or with others of one’s choosing, was preferred over living in communal residential facilities, irrespective of the type of supports available. Whether reflecting on what they valued about their homes or on housing transitions made over the course of experiencing mental health issues, participants highlighted that being in a place of their own was transformative.

Moving into their own places was typically an important turning point in participants’ lives, which were often described as having been “turned upside down” (Maria) leaving nothing except the “stamp of mental illness” (Emma). These turning points included experiences of moving into rental accommodation with outreach support or moving into public housing, each being transformative in that a sense of freedom, space, and privacy was gained. As Elvis described, moving into a place of his own with outreach support seemed to represent a shift from being cared for to doing more for himself, especially cooking in which he took pride, it being connected with his family’s traditions of cooking.

Elvis *There’s a big difference in my life because two years ago, I didn’t have a place of me own. I was living with my mother. And a lot of my time was spent upset, you know, and I wasn’t doing very much. When I moved out of home … basically I got around in a really good way. I did many more things from there on. I spend my time from my house doing all sorts of stuff. For the first year of my house I spent, although I’m still with [outreach team] and all that, I did a lot of cooking for myself, tried to watch as much cooking shows as possible … that became basically an everyday thing … I like to cook a lot of different things to keep myself happy*.

Moving into their own places sometimes also involved a challenging adjustment. For instance, Frank described having been “too scared to move out of [parents’] home” for some time after being “pretty sick”, and reflected that: “when I first moved, I used to get homesick and go home all the time.” Yet, with “good support” from services and family, he had come to appreciate the freedom and privacy to lead his own life: “being able to have a drink and not be pestered. Have a cigarette. Plus me girlfriend comes over … [at weekends], and she brings her old dog” (Frank).

Likewise, after living surrounded by people in a communal residence, Fiona too described moving into her own place as “an amazing adjustment,” which she initially found almost unbearable unless talking to family or friends on the telephone: “I couldn’t bear to be at home. When I was at home, I was in tears. The only time I could be at home would be when I was on the phone.” However, with their support and getting a cat, Fiona described turning this around: “you’d never get me back there! I like my freedom. I like my privacy. I like my peace and quiet now.”

#### It’s Good Around Here: Finding My Niche

Participants highlighted a range of positive qualities in their neighborhoods that improved their sense of being at home where they lived. Hence, descriptions of their neighborhoods often included comments about finding themselves living in a “very friendly street … [where] everybody stops and has a chat” (Annie). But beyond this, some participants also spoke of neighborhood experiences that seemed to foster a sense of finding one’s niche.

For Maria and Emma, women with young families, a place to make a home was not only transformative for themselves and their families, but also supported by experiencing their neighbors as friendly. To illustrate, Maria contrasted the immediate neighborhood of her previous unit on a busy street to her sense of being more accepted and part of her new neighborhood.

Maria *Overall things are improving … it’s hard, but I think it’s better … Moving here—we moved here about six months ago. Being here’s helped a lot. We’re getting to know the neighbours. Where we lived before, [in three years] no one spoke to you hardly. Here, people are friendly—they say hello and we’re respectful of each other. I’ve been in next door and they’ve been over; their son’s come round to play. I’ve got to know the lady over the road, and been supportive of her [when she had some difficulties]. The neighbours are pleased with what we’ve done to the garden too—we’ve fixed it up, planted things, made it neat—the previous tenants trashed the place. … It’s easier for safety reasons too: [daughter] can play without going out on the street and for me not having to go up hill to shops*.

Emma too emphasized having friendly neighbors who helped each other out, some of whom also had children like her:

Emma *I just spend a lot of time looking after the kids and my neighbour’s kids come over and it’s like kids, kids everywhere … it’s good to have like good neighbours … you know, if you need anything or she needs anything, we sort of know we can come to each other and stuff like that*.

Similarly, others described neighbors helping each other out with transport, shopping, or house maintenance as valued aspects of where they lived. Some participants too emphasized that they appreciated living in a familiar neighborhood, or knowing “all the people … it’s like a little country town” (Frank). For instance, seeing “aunties and uncles” and other people whom he knew in the street gave Elvis a sense of connection with others around him, despite his finding social contact more difficult since his illness.

Elvis *I don’t have too much connection with anyone anymore, but I see people, you know. Today I saw [outreach worker] out the front of the supermarket. Everyone’s around me, you know, all live around here … You can see everybody*.

Other participants made similar points in speaking of the positive aspects of getting away from stressful or troublesome neighborhood situations. For instance, speaking of her supported accommodation, Sandra described:

Sandra *It’s safe being here … Nobody can break into your house and do whatever they want with you, so you’re covered. …There’s no drug addicts around here, no alcoholics, everyone’s really nice, so it’s really good*.

Contrasting it with previous experience of neighbors who frightened him because they “trashed” property and gave him “nothing but hell [and] it’s amazing I didn’t have a breakdown,” George too described being much relieved with his present neighbors: “people with, like my own illness, they’re good because we can understand each other … we help each other out, which is good.”

#### Everything’s Accessible Here

The accessibility of local amenities was also represented in participants’ perspectives of home and where they lived. Specifically, access to amenities such as shops, public transport, parks, and services within walking distance of home were noted as an advantage: “The fact that everything’s accessible to me” (Diane). Participants commonly referred to this: “I am living in a good suburb as far as transport goes” [Ron]; and “I’ve got a fair few things I can do in walking distance, plus it’s close to public transport … near shops and everything: the supermarket’s just over the road” (Frank).

Accessible public transport was important not only to get to particular places, but also as a way of getting out and being around people. For instance, Elvis described his sense that public transport could take him anywhere and help him “avoid being sick,” by taking him to places where being around people might distract him from troublesome voices or thoughts.

Elvis *Around my place is everything you could possibly ask for. I’ve got the tram system down the road, which is only about 400 metres away, maybe 500 metres, and I’ve got the train system one street away. I can hear it every day of my life. … Where the tram is, there’s a whole ton of buses that go to K-Mart. They go all sorts of places … And it’s just good to have all that transport around ‘cause I know one day I’m going to get sick…. if I can’t avoid it, I want to get on a bus, I want to get on a tram, I want to do something different*.

In comparison, participants also highlighted how poor housing situations could be challenging, as elaborated below.

### It’s Stressful Living Here

The stressfulness of living in unsatisfactory housing situations worked against participants’ efforts to regain a sense of well-being, while low incomes limited their housing options. For some participants, the difficulties of finding affordable and safe places to rent meant feeling compelled to live in stressful housing situations where “neighbours are quite aggressive and abusive towards me” (Kate) or there were regular disturbances and “other things going on in the flats around me that didn’t contribute to a sense of security and well-being” (Ron). As Ron elaborated:

Ron *One of the things that didn’t help was no job, no financial security, and for a time there, I was really, literally speaking, I was homeless … I wasn’t sleeping on park benches, but I didn’t have a place, which I could call my own, even if it was being rented … there’s no security and yeah, you just live in very dodgy situations. … I don’t want to live in a cheap flat next to a rock band, which is what I’m doing at the moment, [but] I need to have more money to be able to afford to live somewhere else*.

### There’s Few Options

Limited housing options for some participants meant living in “dodgy situations,” as Ron described above. Others described having few alternatives but to live with parents, or to move between friends’ places owing to a restricted income from government income support and restricted access to paid work. For instance, Matt described living “in between friends’ places all the time and I stay a lot at friends’ houses” with mixed feelings: “I feel like I do get in the way … [and] I want to stay there but I don’t want to.” Further, Peter described appreciating that living with his parents had provided a place to live since experiencing mental health issues. Nevertheless, he expressed a sense of loss and missed having his own place: “in some ways it’s like I’m not limited, but in other ways I’m really limited … And I miss cooking, it sort of gets on my nerves … in some ways I’d like to move out so I could cook” (Peter). While not always as stressful as the unsatisfactory housing conditions above, these participants seemed to have a sense of being constrained and were yet to find a sense of being “at home.”

### There’s Poverty, and There’s Powerlessness

Poverty was a reality perpetuated by the necessity to rely on government income support for many participants, whose experiences of mental health issues had disrupted their working lives or marginalized them from the workforce. As Ron described, “my mental illness has created an environment of, you know, it’s placed me in a situation of poverty” (Ron), a situation that could be at least as difficult as mental health issues themselves:

Peter *It’s terrible … economically, the person is living in poverty and that’s a devastating thing … the economic can be emotionally devastating: when you don’t have money, when you don’t have fulfilling work, it can be absolutely devastating … It is actually hard to describe which is worse*.

The predicament of struggling financially also meant that participants were beholden to landlords and bureaucracies for housing and income support, which in turn constrained their power to address challenges related to their living situations: “there’s poverty and there’s powerlessness” (Ron), both of which added to the stressfulness of their living situations.

Participants recounted varied situations involving landlords, housing inspectors, or public housing applications, which led to feeling unsafe at home, frightened, or “overwhelmed” and further held back in their recovery. For instance, having lived in the same rented unit for 8 years, Kate described “my environment’s actually destabilized while I’ve been here” and recounted that whenever her housing and financial security were threatened, such as when the “owner wanted to put [the rent] up by like forty dollars in one hit, which I couldn’t afford … I get so frightened that the only way out I can see is suicide.” For participants, such as Kate, living with a constant sense of vulnerability in their housing underscored the importance of having active support.

Kate *It’s a huge safety net for me … That’s why [support worker]’s trying to get me into housing where I’m not having to deal with estate agents. I mean I’d still have to deal with [housing commission], and sometimes that’s not a good thing either, but it’s more structured … If something goes wrong, you can report it … There’s also the security thing because it’s long term. It’s also because it’s 25% of your wage, so you know if you can’t work, then you know you can afford to live there*.

Hence, despite a supported housing application being declined and the seemingly “indefinite waiting list,” Kate viewed public housing as her most likely way to achieve a sustainable sense of safety and security to move forward with her life. As if to endorse this view having spoken similarly of limited options and being held back, Maria described the restoring of hope and the possibility of having dreams again following moving into her own place in public housing:

Maria *It’s very hard on certain incomes to have those dreams and goals, right, that holds you back. … Our last place you could never buy, whereas this place belongs to the [housing] commission, so there’s a possibility to buy it off them some day. At the moment, it’s hard to put much money away. I try to keep some back for unexpected things…, but even though I’m good with managing money, I can’t seem to save a lot. So maybe buying this place is just a dream, but it’s a possibility*.

### Being Home Is Challenging

Spending time in their home environments was described as challenging by many participants and commonest among those living by themselves (almost half the participants), particularly among women and those outside the workforce. These participants actively struggled with being ill at ease home, so that getting out was helpful in resisting isolation at home. Pets too provided companionship in resisting this isolation.

#### It’s Better Being Out

Preferring to get out rather than spend time at home was described as a strategy for overcoming “being stuck in the house” (George), a sense of being “locked in” (Joan) or “trapped” at home (Kate). This strategy was used most often in contexts of participants not wanting to be on their own, safety concerns, or having a sense of not fitting in where they lived. “Getting out” also required participants, mostly women, to find opportunities for participating and contact with others beyond home. For instance, after years of “doing battle” with depression, Joan described relying on getting out each day:

Joan *I’m out most of the day … that gives me a feeling of, you know, that I’m doing something and I feel happier and I’m not staying home, staying in bed and getting up late and, you know, it’s a chore to get going. …You know, I’m happy to do it. And I’ll go out regardless, unless it’s really raining heavily. I go out every day, Monday to Friday. Yeah, I hate being home, locked in and not getting out*.

For Loretta too, “going out a lot of the day” had become important in helping her feel less gloomy. Even so, the loneliness of her house presented an ongoing struggle to make herself feel safe against the possibility of an intruder: “I’m always frightened someone’s going to break in.” Likewise, to resist fears of being “a sitting target,” during the daytime at least, Kate aimed to go out daily to places where: “I’m around other people…, and I feel a bit more protected” than at home, but also spoke of needing “to be very careful too about people on the street … [because] there’s a few bad characters that hang around” her neighborhood. Participants variously described amenities such as the local library, church, shopping center, a neighborhood community center, or travelling the city by tram as their sanctuaries away from home.

In a different way, getting out of house or neighborhood was also a strategy for dealing with a sense of feeling “the odd one out” (Janis), or not having found one’s niche, and the ensuing sense of isolation:

Janis *You do get cabin fever round here, you need to get out of the area … To get a bit of alternative culture and life and see gay people, you really need to go somewhere like that to feel grounded … to feel grounded in your sexuality and being in a community and stuff like that, you really need to do that every couple of weeks… ‘cause out here you feel really isolated and that’s a really big issue*.

#### It’s Not Four Walls

For participants who described being home as challenging, getting a cat or dog had been instrumental in turning a place to live into a home and making life easier, as Kate described.

Kate *I’m a lot happier now that I have a cat … She’s made a huge difference to me ‘cause when I used to come home, I used to try … I’d have to be in someone else’s place, like I couldn’t be on my own. Since I’ve had her, I’m not as bad. You know, she’s my baby and I just want to be with her. …And she does funny things. She makes me laugh sometimes. It brings you out when you’re feeling down in the dumps. …it’s great to come home to someone, well or come home to a cat. … I know there is something waiting for me here. It’s not four walls*.

For others, pets were special companions at home and meant not “being stuck in the house” without company (George): “like I’ll lie on the couch, he’ll come up and lie beside me, or if I’m down, he knows, he’ll come jump on my lap and start licking my face to cheer me up.” As Janis further elaborated:

Janis *It’s like the place just seems empty when [my dog]’s not here. … I know she’s only a tiny little thing but just having her running around, or jumping up on the couch with me. It’s just this constant companion you know and when she’s not here, the place is just desolate*.

### Balancing Self-Determination and Need for Support

When talking about their support workers, participants described different experiences, ways of viewing and using the available support. Valuing support as well as autonomy and self-sufficiency meant also some ambivalence regarding the need for support workers. Mostly, participants valued a helping hand from services to navigate difficult times and transitions. For example, Peter described community mental health staff as supporting him to transform a “very unhappy life” with “a helping hand to pull me out of that nightmare” (Peter), and in Diane’s words: “really without them you just couldn’t get through it.”

Qualities emphasized in this kind of support were a sense of genuine caring and service providers doing their best to help; and service providers being people who participants could relate to and who were respectful: “[she] treated me like a human being, treated me like a real human being” (Felicity). This included staff who valued their perspectives and worked *with* participants. As Janis elaborated:

Janis *It is really good that she [outreach support worker] takes me out ‘cause I don’t have to worry about concentrating and we can go to places that I might not be able to take myself … Because of the drugs and everything, I can’t concentrate a long time. Yeah, so [she] is great like that. …she’s really helpful too when I can’t drive at all and she’ll take me grocery shopping or whatever. And when I get out of hospital, like going back into the supermarket and things like that, it’s really hard. … she’ll go with me and get me back on my feet*.

Furthermore, as Elvis illustrated, his almost daily contact with staff of an assertive outreach team helped him to keep the voices at bay and gave him practical strategies for getting through the day:

Elvis *That’s one of the biggest structures in my day. If I didn’t have [outreach team], I’d be pretty loony. I wouldn’t be very healthy. …It’s nice to hear from them, you know. It’s someone to talk to. It’s stopping the voices. If you gettin’ really bad, you can tell them about it. … He [case manager] kind of regulates you. He says, ‘I’m going to send you to a park, I’m going to send you on a walk.’ And he just keeps you [going], he’s pretty good that way … I like him a lot*.

Support workers were seen too as creating conditions in which participants could go forward with rebuilding their lives or more like mentors. For instance, as Maria described, she had been encouraged by her support worker to rediscover thinking of herself as a person: “You’re Maria with the mental illness, you’re not just a mental illness” and had learned to view her support worker as a resource:

Maria *It’s taking the staff’s wisdom … taking it in and on board and applying it as much as I can to my life … learning [from my support worker] that I had dreams as much as she’s got dreams. …We’ve come a long way me and [support worker] from me not just looking at her as a staff, and just a person that’s there that gets a wage and that’s it, and what do they really care? They’ve got everything, and I’ve got nothing, and what do they really care? …to yeah, they’re doing a job but really using their knowledge and wisdom … getting as much as I can out of them. Like okay, I’m not here to bag ‘em, I’m here to learn how they talk. I’m here to learn how they say I like and I choose and I am*.

Conversely, from participants’ perspectives, service providers sometimes seemed either to underestimate or misunderstand the place of “keeping things settled” when they appeared to be doing well, in order to support moving forward with their lives. For instance, with three years of unsettled and difficult times behind her, Emma described:

Emma *I was going along alright and then the doctor told me I was gonna be discharged from the [outreach] service and then I went downhill … she’s done it twice, like tried to discharge me, and both times I’ve got sick. … what has been known for me is, like with stress and change and stuff like that, I just go down. … I’ve been through a lot over those years, like changes … So yeah, I’ll just stay where I am I think, until I’m sort of really comfortable*.

Similarly, Julie described “everything’s settled down” after some years of upheaval in her life, being keen to “keep things stable” and not push herself too quickly: “I just feel really content at the moment, the way things are. I’m not going to push myself to the next step or anything, just stay the way I am at the moment.”

## Discussion

Findings from this study align with the well-documented preferences of the majority of consumers to live in their own housing, and with persons of their own choosing (6). They are also consistent with previous research indicating the value of establishing a place of one’s own for the sense of freedom, personal space, and privacy gained, and in supporting recovery (2, 17, 36). Wide-ranging personal and contextual factors contributed to participants’ experiences of their housing, with feeling at home in their own house and in their neighborhood each seeming important. Indeed, home held many of the same meanings for people experiencing mental health issues in this study as widely held in communities, as noted elsewhere (6, 37). That is, their homes signified personal space, security, privacy, a refuge, and freedom to pursue their own interests and activities. A “home” is likely to be particularly potent for formerly homeless people (13, 37), yet, the desire for these elements of a home was both tangible and difficult to secure for those participants in this study who, for lack of other options, lived with parents and in transient living arrangements.

### Transitions

Moving into their own places, whether in public or rented housing, was typically transformative in participants’ lives and supports the view that housing is an influential factor in the process of recovery (36, 38). Previous qualitative metasyntheses too have suggested getting a home can be a positive turning point (17) and an important base from which to rebuild one’s life (2). Housing transitions are also known to be more frequent among people with persistent mental illness compared to the general population (11). While the majority of participants in this study had been in their current housing for 2 years or more, their experiences of housing transitions were diverse and variously followed an inpatient stay, managing homelessness, living in a supported group accommodation, or living with parents as the consequence of having been unwell. As Krotofil et al. (6) noted, experiences of specialist mental health staffed supported accommodation that emphasize moving on may signify growth, opportunity, and support recovery, but time-limited accommodation may not only be experienced as creating disruption, uncertainty, and stress but may also work against human needs for security and familiarity (39). For participants in this study, the freedom, space, and privacy of their own place were transformative irrespective of whether they had moved from a residential service setting or their parents’ home and whether their home was a temporary or ongoing housing arrangement.

Lived experiences of housing are more dynamic than a focus on either being housed, moving out of hospital, or from homelessness to housing might suggest (37). Hence, as participants in this study illustrate, experiences of being housed and making a home need to be understood within an ongoing life story, in which significant disruption or displacement may have occurred. Less has been written about lived experiences of moving between living situations over time than experiences of transitions to community living following an inpatient stay. Nevertheless, a recent systematic review of research on the latter by Mutschler et al. (40) highlighted several conditions necessary for transition that were also highlighted by participants in this study. Specifically, in common with participants in this study, Mutschler et al.’s review emphasized the importance of safety, supported autonomy, and opportunity to engage in activities that support connection to others in one’s community. Also consistent with Mutschler et al.’s (40) review, participants in this study described moving between living situations as daunting and presenting varied challenges related to having limited financial resources, living in poverty, and interpersonal challenges in one’s immediate living environment. In addition, participants described struggling and active efforts to improve their housing situations that were similar to the hard work reported by mental health consumers seeking housing assistance in an Australian study by Honey et al. (1). Family members, case managers, and outreach support workers were all noted too as crucial ongoing supports in facilitating ultimately successful transitions.

### Neighborhood Experiences

The findings of this study highlight the power of neighborhood experiences to contribute to individuals feeling at home, making a home life, and supporting recovery. Strikingly, a sense of familiarity or longstanding connection with a neighborhood seemed to enhance participants’ sense of being at home where they lived, as did proximity to amenities such as public transport, shops, and opportunities for interactions with other people. This supports the view that further exploration of how people relate to places, as well as the resources available to them locally, are necessary to understand the relationships between place and health (41). Further, settling into neighborhoods experienced as safe, and in which encounters with friendly, accepting, and respectful neighbors occurred, were transformative. This is consistent with previous research indicating that positive neighborhood relations and perceived neighborhood safety are important to individuals feeling that they belong, are accepted, and to their well-being (42). It also suggests the role of “place” merits further consideration in research and practice informed by recovery frameworks (43, 44). Furthermore, beyond neighbors providing informal supports, opportunities for reciprocity in care and support were evident in participants’ stories of positive interactions with neighbors, perhaps most notably for parents with young children.

As in previous housing research, participants in this study reported both positive and negative experiences of interactions with neighbors (1, 45). The neighborhoods of people experiencing persistent mental health issues have previously been reported to be of poorer physical quality, and to have higher levels of crime than other neighborhoods in Australia and elsewhere (10, 46). This is particularly an issue in Australian neighborhoods dominated by social housing (1). Not surprisingly then, safety and security are reported as prominent concerns in urban settings (47), with poor neighborhood relations including threats from neighbors and strangers and perceived lack of safety thought to be important in accounting for distress (48). As well as hostile interactions with neighbors, participants in this study reported interactions with estate agents, landlords, and housing services as challenging or intimidating and a source of additional stress. Consistent with Honey et al.’s findings, social and economic disadvantage associated with mental illness were key reasons that participants sought housing assistance and support, but they also felt disempowered by the authorities and bureaucracies on which they were reliant. This points to the need for new ways to interact with and support people seeking assistance in relation to their housing and financial situations, which promote feelings of safety and security rather than undermining them. For instance, participatory approaches might involve the peer workforce to develop housing supports that address locally relevant needs for information and support related to tenants’ rights, access to effective advocacy, and assistance in navigating issues with neighbors, landlords, and housing and welfare bureaucracies (1).

### Strategies for Resisting Social Isolation and Loneliness

Lived experiences of loneliness are reported across communal and individualized housing settings (6), and findings in this study align with previous research highlighting that living by oneself can be challenging (2). Few other studies have identified specific strategies used by people experiencing mental health issues to manage these situations, yet participants in this study identified active strategies for managing living by oneself and being ill at ease when at home. One recent Canadian qualitative study by Piat et al. (49) reported that capacity to reach out to others, engage with family, and keep busy were strategies used to manage loneliness by tenants living in housing with outreach support. In comparison, the strategies of participants in this study centered on getting out of the house to be around other people, rather than solely for the company of friends or family. Hence, participants in this study appeared to actively use their time in ways that supported self-managing their living situations by seeking out community arenas where other people were likely to be encountered. At the same time, use of this strategy was dependent on access to these arenas or available public transport for getting out and being around people, factors not necessarily routinely considered in planning and organizing housing support services or the actual location of social housing.

Pet ownership too afforded more ease at home for participants in this study living by themselves, a number of whom highlighted their pets as having transformed their capacity to be at home. The ways in which pets provide companionship, emotional comfort, and support in assuaging feelings of sadness, loneliness, and upsetting experiences, and sometimes create a bridge for making social connections in neighborhoods have been reported elsewhere (50–52). More rigorous research is needed to better understand how pets contribute to mental health (50). Nevertheless, the importance of pets in the home lives of people experiencing ongoing mental health issues may be under-acknowledged as a source of support for mental well-being, and consideration of pets needs to be routinely part of collaborative care planning in mental health services (53).

### Implications for Supports

Evidence from this and previous research points to the need for greater focus on the provision of housing with outreach support to align housing services with consumer preferences and recovery-oriented practice principles, so that opportunities to have a home, with its associated potential for achieving well-being, are made more widely accessible. In comparison to research on housing preferences, housing characteristics, and their relationships to consumer outcomes, the nature of the outreach support in practice is relatively under-researched and its most effective components in need of more rigorous research. The findings of this study underscore previous reports that indicate support workers who demonstrate care and respect for the person are valued, together with support that is collaborative and provides practical assistance personalized to the individual’s situation (2, 10, 39, 54). Further, these findings extend understanding of what is helpful in housing-related outreach support and might be evaluated in future studies. Specifically, the importance of information and support to navigate issues with neighbors, landlords, and housing bureaucracies is highlighted. Supports could usefully extend to addressing neighborhood concerns, harnessing natural supports and opportunities in communities for getting out to places with possibilities for being around and interacting with other people (8, 10, 39). This type of practice may be constrained by how the scope of housing-related support is understood within services, and require additional resources to facilitate progress (39). Nevertheless, to promote satisfaction with housing and well-being, emerging evidence suggests designing housing services in such a way as to facilitate opportunities to engage in satisfying occupations, social interaction, and to access information and support is important (8).

This research also underlines the need for workers to flexibly adjust the support provided to respond to the housing-related challenges faced by people experiencing mental health issues while also fostering their autonomy (39, 55). In addition, the impacts of potentially losing either the safety net provided by income support or ongoing housing support deserve better recognition as factors undermining stability in housing and well-being. For people with ongoing mental health issues, poverty and social exclusion co-exist and make each other worse (56), so that both need further research to better reduce their impacts on individuals’ everyday lives and well-being.

### Limitations

This study may be considered limited by being located in metropolitan Melbourne, albeit that it included participants living in diverse neighborhoods from inner city and outer urban suburbs. Nevertheless, they may not be representative of the nature of housing and support services available in the urban areas of other Australian cities, or internationally. Likewise, all participants in this study had access to mental health services, so that the views of people experiencing mental health issues who are not in contact with services are also not represented. Similarly, the findings do not include the experiences of residents of supported group accommodation. Further, men and women appeared to speak somewhat differently about their housing experiences in that men tended to frame their struggles to secure satisfactory housing in relation to their efforts to rejoin the workforce or to seek better paid employment, while women described day-to-day struggles in relation to family and raising children in greater detail. However, there was insufficient data on these topics to interpret them as gendered issues. Future research could usefully attend more closely to how gender and other social attributes shape lived experiences of housing and neighborhoods, so as to design supports that are responsive to diversity in people’s needs and concerns.

The study also has a number of strengths. The creation of conditions for listening, fostering dialogue, and working together are critical processes in participatory research (19), so that the extent of engagement with people experiencing mental health issues was pivotal in keeping this research closely connected with a consumer perspective of the issues being explored. Specifically, the participatory reference group provided a space for dialogue, decision-making, debriefing, reflection, and interpreting the findings, which served to enhance authenticity in representing participants’ views. In addition, follow-up interviews enabled a collaborative member checking process, whereby individual participants reflected on their stories with the first author.

## Conclusion

Drawing from participatory research undertaken with people experiencing mental health issues living in an Australian urban community, this paper illuminates housing and neighborhood experiences that contributed to or challenged participants’ sense of being “at home” where they lived. The findings underline that lived experiences of being housed and making a home can be transformative processes; they also highlight active efforts and strategies used by people experiencing mental health issues that warrant further research. Given the significance of pets in people’s lives, recognition of pets in care planning is suggested. The findings provide insights into how the possibilities for feeling “at home” were contextualized by participants’ experiences of neighborhoods. This underscores that more explicit attention to neighborhood safety and access to amenities in the development of housing options, and to harnessing supports for people experiencing mental health issues to navigate issues with neighbors and housing are each required. Furthermore, it highlights that the role of “place” in facilitating recovery merits further consideration in research and practice.

## Data Availability Statement

The datasets generated for this study are not available. Ethical approval for wider sharing of the datasets was not granted in the interests of protecting participants’ privacy and confidentiality.

## Ethics Statement

This study involving human participants was reviewed and approved by the Human Research Ethics Committees of The University of Melbourne (HREC 020595), La Trobe University (FHEC03/070), and North Western Mental Health (E/02/001). The participants provided their written informed consent to participate in this study. Written informed consent was obtained from the individual(s) for the publication of any potentially identifiable images or data included in this article.

## Author Contributions

EF designed the study, with guidance from the co-authors (CH, FM) and the participatory reference group (also known as the Participating Lives Project Reference Group), of which CH and FM were members. EF interviewed participants and analyzed the data, informed by the participatory reference group perspectives. EF drafted the manuscript. CH and FM reviewed, revised, and added material to manuscript drafts.

## Funding

This study was supported by an OT AUSTRALIA Research Award, and a Faculty of Health Sciences Research Grant from La Trobe University.

## Conflict of Interest

The authors declare that the research was conducted in the absence of any commercial or financial relationships that could be construed as a potential conflict of interest.
